# Systemic inflammation induced by lipopolysaccharide aggravates inherited retinal dystrophy

**DOI:** 10.1038/s41419-018-0355-x

**Published:** 2018-03-02

**Authors:** Agustina Noailles, Victoria Maneu, Laura Campello, Pedro Lax, Nicolás Cuenca

**Affiliations:** 1 Physiology, Genetics and Microbiology, University of Alicante, Alicante, Spain; 2 Optics, Pharmacology and Anatomy, University of Alicante, Alicante, Spain

## Abstract

Retinal neurodegenerative diseases involve a scenario of inflammation and cell death that leads to morphological alterations and visual impairment. Non-ocular inflammatory processes could affect neurodegenerative retinal disorders and their progression, at least in part by activating microglial cells and releasing pro-inflammatory cytokines. Our purpose was to study the consequences of a systemic inflammatory process in the progression of retinal degeneration in P23H rats, a retinitis pigmentosa (RP) model. In order to induce a mild chronic systemic inflammation, we administered low doses of lipopolysaccharide (LPS) from age P20 to P60 to dystrophic P23H rats and healthy SD rats. Visual responsiveness was assessed by electroretinography (ERG). The morphological state of the retinas was analyzed by fluorescent immunohistochemistry (IHC), evaluating the number, morphology, and connectivity of different neuronal populations by means of cell type-specific markers. Microglia density, distribution, and degree of activation were evaluated by IHC and flow cytometry. The expression levels of inflammation- and apoptosis-related genes were analyzed by qRT-PCR arrays. Low-dose LPS administration did not induce significant functional or morphological changes in the retina of SD rats, although at the molecular level, we detected expression changes in genes related to apoptosis. Otherwise, systemic injection of LPS into P23H rats induced a further deterioration in the ERG response, with greater loss of photoreceptors and worsening of synaptic connectivity, accompanied by increasing numbers of microglial cells, which also showed a more intense activation state. Several inflammation- and apoptosis-related genes were upregulated. Our results indicate that chronic exacerbation of the inflammatory response in response to LPS accelerates neurodegeneration in dystrophic P23H rats, suggesting that in patients with ocular neurodegenerative diseases, peripheral damage, as a systemic infection or chronic inflammatory process, could accelerate disease progression, and should be taken into account in order to select an appropriate therapy to revert, block or slow-down the degenerative process.

## Introduction

Retinal neurodegenerative diseases lead to morphological and functional impairment within a scenario of inflammation in which the three main cell death pathways (apoptosis, necrosis, and autophagy) converge^1–4^. Microglia activation and elevated levels of pro-inflammatory cytokines are a common feature in neurodegenerative disorders, such as Parkinson’s^5,6^, Alzheimer’s^7,8^, and Huntington’s diseases or amyotrophic lateral sclerosis^9^. Activated microglia release inflammatory mediators steadily over time, which boosts the inflammatory cycle^10^. Unlike the protective role of acute neuroinflammation, chronic neuroinflammation mostly damages nervous tissues^11^. Also, retinal neurodegenerative diseases, such as retinitis pigmentosa (RP)^3^, age-related macular degeneration^12^, and glaucoma^13,14^ are concomitant with chronic microglial activation and neuroinflammatory process. Retinal microglial cells may also be activated by systemic infection by fungus or virus^15,16^ and by immunosuppression or peripheral inflammation^17^, which could worsen the degenerative process. However, the mechanisms by which systemic inflammation and microglial activation exert their effects on chronic neurodegeneration are not yet widely understood.

Lipopolysaccharide (LPS) administration is commonly used as a model of neuroinflammation^18–20^. LPS induces microglial activation by a Toll-like receptor 4 (TLR4)-dependent pathway, and is accompanied by the production and release of pro-inflammatory cytokines, such as interleukin (IL)-1β, IL-6 and Tumor necrosis factor alpha (TNF-α), by the Mitogen-activated protein kinase (MAPK) and Nuclear factor kappa beta (NF-kβ) routes^21,22^. Systemic administration of LPS also activates retinal microglia^23,24^, but the exact effect of LPS-activated microglial cells on photoreceptor death and the mechanisms underlying microglia–photoreceptor crosstalk remain to be determined.

The main objective of this work was to elucidate whether a peripheral inflammatory process must be considered a risk factor in the progression of retinal degenerative diseases. We studied the consequences of systemic administration of LPS in P23H rats, an animal model of RP. We decided to administer low doses of LPS in order to trigger the alert status of microglia, but avoid a massive influx of pro-inflammatory cells to the retina, in an attempt to mimic a mild chronic peripheral condition. Our data suggest that peripheral inflammation exacerbates the inflammatory response in the retina and accelerates neurodegenerative events in the P23H rat model, causing greater loss of photoreceptor numbers and increased retinal dysfunction.

## Materials and methods

### Animals

Homozygous P23H line 3 rats, kindly provided by Matthew LaVail (UCSF School of Medicine; www.ucsfeye.net/mlavailRDratmodels.shtml), were used as a RP model. Sprague–Dawley rats (SD) were used as a healthy control group (Harlan Laboratories, Barcelona, Spain). Twelve SD rats and 12 P23H rats were housed in cages under controlled photoperiod (12-h light/12-h dark), humidity (55–60%), and temperature (23 ± 1 °C) conditions. Both food and water were provided *ad libitum*. All procedures received prior approval from the ethics committee for animal care and use at the University of Alicante (UA-07/22/2013). The animals were treated according to current guidelines and regulations for the use of laboratory animals (NIH, ARVO and European Directive 2010/63/EU), in an effort to minimize their suffering and limit the number of animals used.

### LPS administration

LPS was purchased from the Sigma-Aldrich laboratory in St. Louis, MO (USA). A stock solution of 400 µg/ml was prepared in saline and stored in aliquots of 1 ml at −20 °C until administration. A total of six SD and six P23H rats received three weekly injections of LPS intraperitoneally (i.p.) (60 µg/kg in saline) (LPS-injected rats), whereas six SD and six P23H rats were used as control groups receiving saline injections at the same time points (vehicle-injected rats). Treatment began at P20 and continued until P60.

### Electroretinographic (ERG) records

ERGs were performed under scotopic conditions at P60, after completion of the treatments. Scotopic flash-induced ERG responses to light stimuli produced by a Ganzfeld stimulator were recorded in both eyes. Light stimuli were administered for 10 ms at 11 increasing luminances, ranging from −5.2 to 0 log cd s/m^2^. Between 3 and 10 consecutive recordings were averaged for each light level. A 10-s interval was provided between flashes in the case of dim flashes (−5.2 to −1.4 log cd s/m^2^), and up to 20 s for the brightest flashes (−0.8 to 0 log cd s/m^2^).

### Immunohistochemistry

Histological studies were conducted at P60. Vertical cryostat sections were prepared for immunostaining following procedures that have been well-established in the literature^25,26^. The sections were incubated overnight with the primary antibodies: monoclonal mouse anti-Bassoon (1:1000; Enzo Life Sciences, Plymouth Meeting, PA, USA), polyclonal rabbit anti-GFAP (1:50; Dako, Santa Clara, USA), polyclonal rabbit anti-cone arrestin (1:500; Millipore, Billerica, MA, USA), polyclonal rabbit anti-calbindin (1:500; Swant, Bellinzona, Switzerland), polyclonal rabbit anti-Iba1 (1:1000; Wako Chemicals, Richmond, VA, USA), or monoclonal mouse anti-MHC class II RT1B (clone OX-6, 1:200; AbD Serotec, Kidlington, UK). The secondary antibodies employed were either Alexa Fluor® 555/488 anti-mouse or anti-rabbit IgG (1:100; Molecular Probes, Eugene, OR, USA). Images were taken using a Leica TCS SP2 confocal laser-scanning microscope (Leica Microsystems). Images from SD and P23H sections were parallel processed with Adobe Photoshop 10 software (Adobe Systems Inc., San Jose, CA, USA).

### Quantification of microglial markers

To analyze microglial cells in each group of animals, we examined vertical retinal sections. The quantity of cells expressing either or both markers (Iba1+/MHC-II−, Iba1−/MHC-II+ and Iba1+/MHC-II+) was determined at ×63 magnification. The data represent counts for the total length of the section analyzed and were then extrapolated to 1 mm of retinal tissue (as obtained through the ImageJ software; National Institutes of Health, Bethesda, MD, USA). We analyzed at least two non-consecutive retinal sections of each animal belonging to each experimental group, including the optic nerve, and the counts are those for the total length of each retinal section.

### Quantification of gliosis in Müller cells and astrocytes

To assess the response of Müller cells and astrocytes to LPS administration, we quantified gliosis in Müller cells and astrocytes using anti-glial fibrillary acidic protein (GFAP) antibodies. To do that, we analyzed four animals per experimental group, evaluating in each retinal section four equidistant regions in the temporal-nasal axis: two in the temporal area (temporal-peripheral and temporal-central) and two in the nasal area (nasal-peripheral and nasal-central). Gliosis in Müller cells and astrocytes were evaluated by measuring the fluorescence area related with GFAP immunostaining using the ImageJ software.

### Quantification of photoreceptor rows, synaptic connectivity, and structural integrity of cone photoreceptors

To study retinal degeneration, we quantified photoreceptor rows, using the nuclear marker TO-PRO 3, in at least two non-consecutive retinal sections of each animal. Quantifications were performed in six equidistant regions in the temporal-nasal axis: three in the temporal area (temporal-peripheral, temporal-medial, and temporal-central) and three in the nasal area (nasal-peripheral, nasal-medial, and nasal-central). An average value of photoreceptor rows was calculated for each animal.

We also evaluated synaptic connectivity in the outer and inner plexiform layers (OPL/IPL) of the retina by measuring the fluorescence area associated with Bassoon immunostaining using the ImageJ software. In all sections analyzed, this quantification was performed close to the optic nerve. The structural integrity of cone photoreceptors was evaluated by immunostaining with a polyclonal rabbit anti-cone arrestin antibody.

### Flow cytometry

To identify microglial population and cell activation by flow cytometry, a retinal cell suspension was subjected to a triple labeling with a cocktail of antibodies: anti-CD11b conjugated to APC (allophycocyanin—Blue, clone M1/70; eBioscience), anti-MHC Class II PE conjugated (phycoerythrin—Red, clone M5/114.15.2; Miltenyi Biotec, Bergish Gladbach, Germany) and anti-CD45 FITC conjugated (clone 104.2, Miltenyi Biotec). Each rat retina was analyzed individually. Data were acquired on a LSRFortessa flow cytometer (BD Biosciences) and analyzed using FACSDiva software (BD Biosciences).

### qRT-PCR arrays

To analyze the mRNA expression levels of genes associated with apoptosis and inflammation pathways, we used two commercial arrays from Qiagen® (Custom RT^2^ PCR Apoptosis array 96 wells; Qiagen, Hilden, Germany) and Applied Biosystems (TaqMan® Inflammation Array 96 wells FAST plates; Applied Biosystems, Carlsbad, CA, USA). Total RNA from retinas of vehicle- and LPS-treated animals was obtained using the mirVana total RNA Isolation kit (Ambion Ltd., Cambridgeshire, UK) and treated with Turbo DNase (Ambion Ltd). Total RNA quantification was carried out using a Nanodrop-1000 device (Thermo Fisher Scientific, Waltham, MA, USA). Two hundred nanograms of total RNA from each sample were reverse transcribed into complementary DNA (cDNA) using the RT^2^ first strand kit (Qiagen) and High Capacity RNA-to-cDNA Kit (Applied Biosystems). We quantified the mRNA expression levels of *TNF*-*α*, *IL-1α*, *IL-1β*, *Caspase-1*, *Caspase-8* and *CX3CL1*, *p53*, *Bcl-2*, *Bad*, *Bax*, *Hrk*, and *Apaf-1*, *GSK3β*, *Akt* and *mTOR*. *RNA18S*, *Actβ*, *Hprt1*, and *Rpl13α* were used as house-keeping genes. *RNA18S* was used to normalize the analysis. Each run also included negative controls. A StepOnePlus® system (Applied Biosystems) was used to conduct PCR experiments. The data analysis was performed using the ΔΔCt based calculations using free array data analysis web portal software, provide by Qiagen®. Data obtained from Applied Biosystems arrays were analyzed using the *ExpressionSuite* software (www.thermofisher.com/es/es/home/technical-resources/software-downloads/expressionsuite-software.html) with the same calculation method.

### Statistical analysis

SPSS 20.0 software (IBM, Armonk, NY, USA) was used to perform the statistical analysis and a Multivariate Analysis of Variance (MANOVA) was calculated to assess the effects of LPS administration. A Bonferroni’s test was used to perform post-hoc pairwise comparisons when the significance level was found to be 0.05. All the analyzed categories exhibited normal distributions and homogeneity of variance. Statistical significance was established at *P* < 0.05. The data were subsequently plotted as the mean ± SEM.

Flow cytometry results were analyzed using Student’s *t*-tests to compare values in different animal groups. Results were expressed as the mean ± standard deviation and were considered to be statistically significant at *P* < 0.05.

## Results

### The effects of systemic LPS administration on ERG retinal responses

**Fig. 1:**
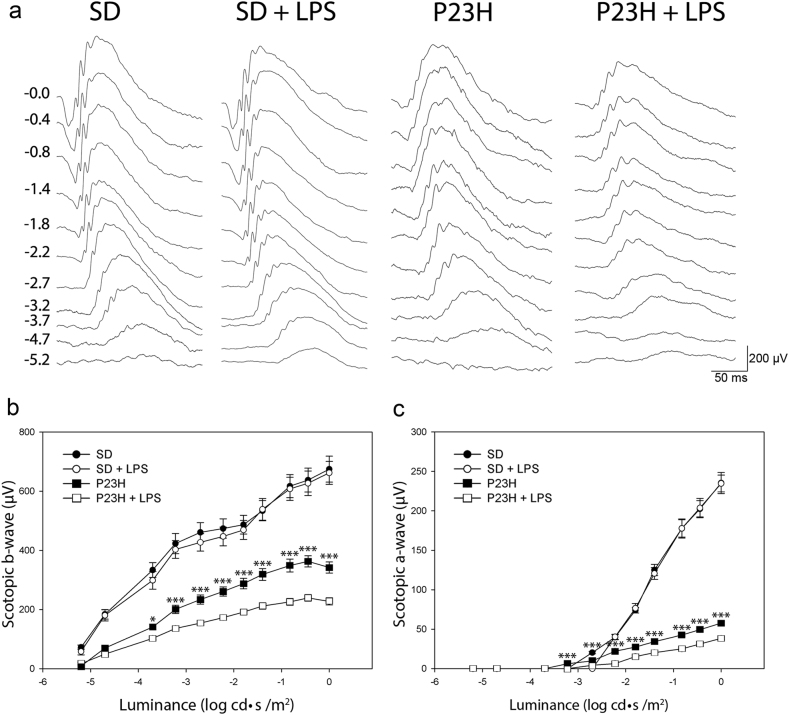
Effect of LPS on functional changes associated with retinal degeneration. **a** Representative scotopic full-field ERG waveforms from P60 SD and P23H rats injected with vehicle or LPS from P20 to P60. Units on the left show input flash intensities in log cd s/m^2^. **b**, **c** Luminance-response curves for mixed scotopic b-waves **b** and a-waves **c** from vehicle-injected SD rats (black dots), LPS-injected SD rats (white dots), vehicle-injected P23H rats (black squares), and LPS-injected P23H rats (white squares). Data are represented as mean ± SEM, *n* = 6. *P < 0.05, ***P < 0.001; ANOVA, Bonferroni’s test

To assess the effect of low-dose systemic LPS in the functional activity of the retina of P23H and SD rats, ERG responses induced by scotopic flash were recorded in four animal groups (vehicle-injected SD, LPS-injected SD, vehicle-injected P23H, and LPS-injected P23H; *n* = 6 in each group). As shown in Fig. 1, chronic administration of LPS to P23H rats reduced the ERG response to light flashes. The maximum a- and b-wave amplitudes registered were 31% and 33% lower (respectively) in LPS-injected P23H rats than that observed in vehicle-injected dystrophic animals (57.7 ± 1.7 μV and 38.3 ± 1.2 μV for the a-wave, 342.2 ± 5.1 μV and 227.7 ± 4.5 μV for the b-wave; analysis of variance (ANOVA), Bonferroni’s test, *P* < 0.001, for both cases; Fig. 1). The same doses of LPS did not affect the retinal response of SD rats.

### The effects of systemic LPS administration on the number of photoreceptor rows, structural integrity of cone photoreceptors, and synaptic connectivity

**Fig. 2:**
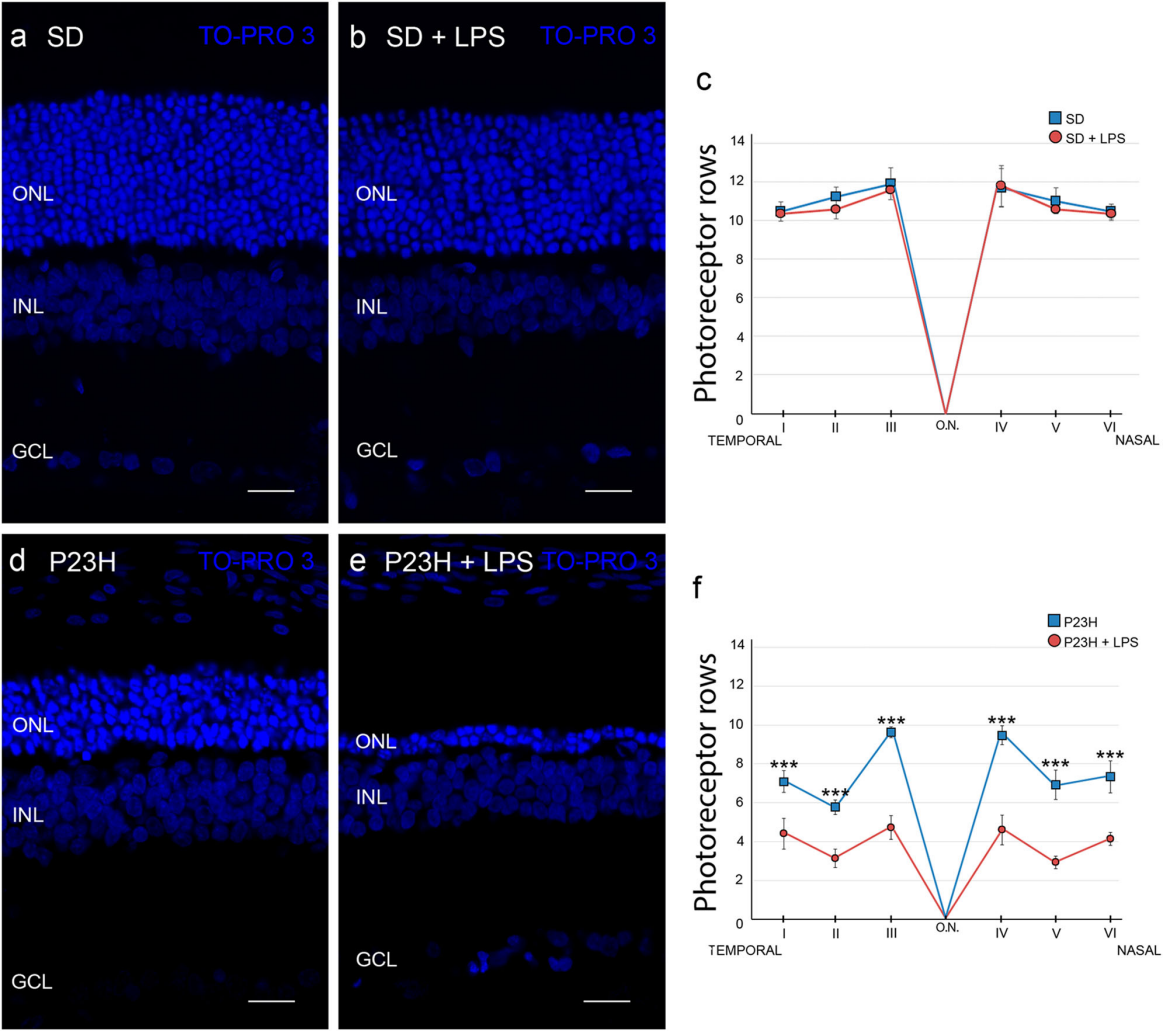
Effect of LPS on the number of photoreceptor rows. Representative images of vertical retinal sections from SD **a**, **b** and P23H rats **d**, **e** injected with vehicle **a**, **d** or LPS **b**, **e**. Nuclei were stained with TO-PRO 3 (blue). **c**, **f** Average number of photoreceptor rows along central sections of the retina in vehicle- and LPS-injected SD rats **c** and P23H rats **f**. Data are represented as mean values ± SEM, *n* = 6 in each condition. ***P < 0.001; ANOVA, Bonferroni’s test. ONL outer nuclear layer, INL inner nuclear layer, GCL ganglion cell layer. Scale bar, 20 μm

The analysis of retinal sections of each animal group stained with TO-PRO 3 revealed that the retinas of vehicle-injected P23H animals had an average of 7.7 ± 1.1 rows of photoreceptors, whereas vehicle-injected SD rats showed an average of 11.1 ± 0.8 photoreceptor rows (Fig. 2). The systemic administration of LPS did not induce a significant reduction of photoreceptor rows in SD rats, whereas the same LPS dose reduced by 45% the number of photoreceptor rows in P23H rats (4.2 ± 0.8 rows; ANOVA, Bonferroni´s test, *P* < 0.001). This reduction in the quantity of photoreceptor rows was evident in all retinal sections analyzed (data not shown).

**Fig. 3:**
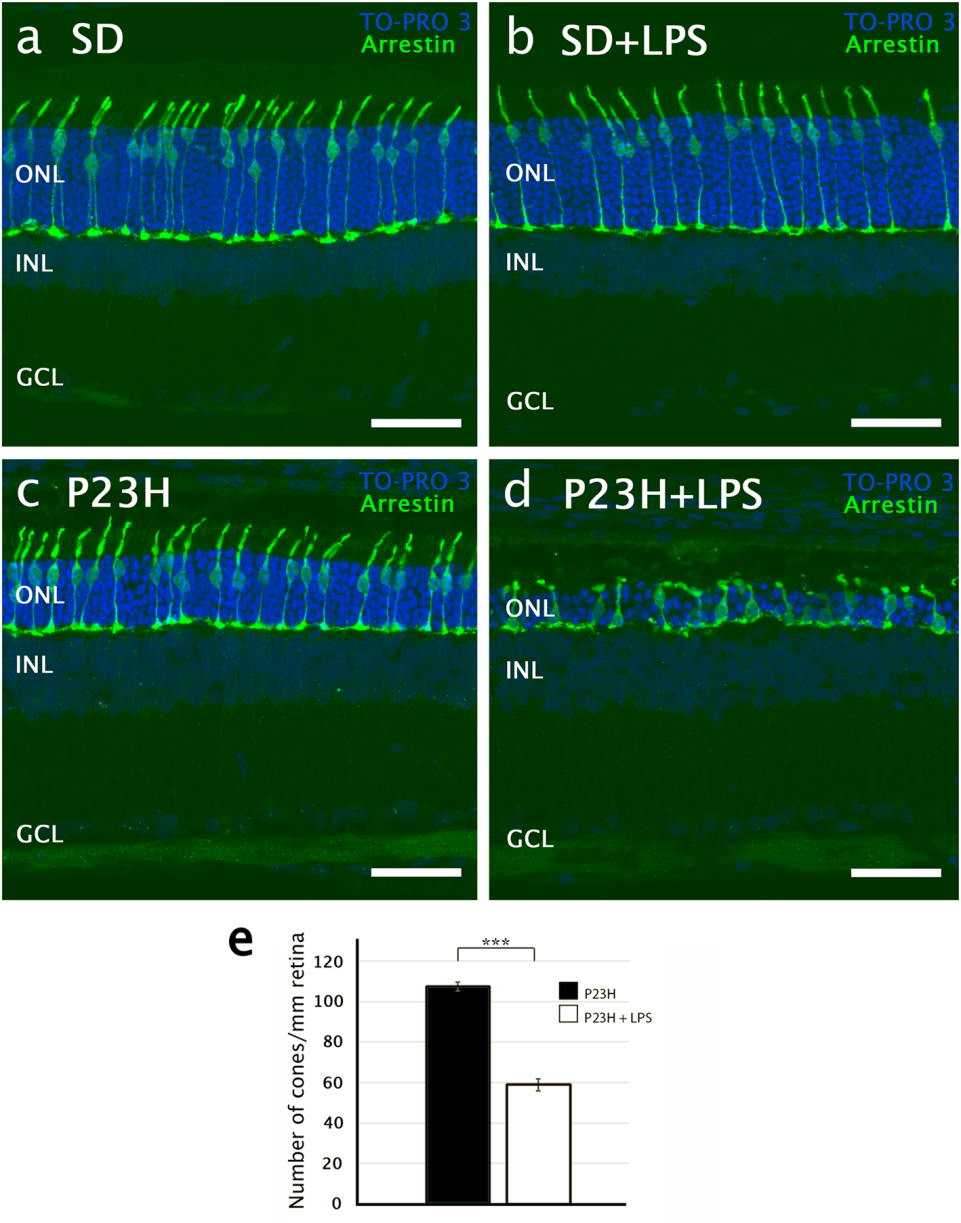
Effect of LPS on the number and integrity of cone photoreceptors. **a**–**d** Confocal images of representative vertical retinal sections showing the cone morphology after immunostaining with antibodies against cone arrestin (green) in SD **a**, **b** and P23H rats **c**, **d**, vehicle-injected **a**, **c** and LPS-injected **b**, **d**. Nuclei were stained with TO-PRO 3 (blue). **e** Quantification of cone photoreceptors per mm of retina in P23H vehicle- and LPS-injected rats. Data are represented as mean values ± SEM, n = 6. ***P<0.001; ANOVA, Bonferroni’s test. GCL ganglion cell layer, INL inner nuclear layer, ONL outer nuclear layer. Scale bar, 40μm

We also evaluated the number, shape, and structural integrity of cone photoreceptors in the retinas of each experimental group, using antibodies against cone arrestin. In SD rats, the administration of LPS did not provoke significant differences in morphology or number of cone photoreceptors (Figs. 3a, b). In vehicle-injected P23H rats, cones showed inner and outer segments than were shorter than those observed in SD rats (Figs. 3a, c). But cone photoreceptors experienced more drastic changes in LPS-treated P23H rats. In these animals, the cones were very small, with short and swollen outer segments (Fig. 3d). The axons were missing entirely, and the pedicles emerged directly from the cone cell bodies. The number of cone photoreceptors was also smaller in LPS-treated P23H rats than in vehicle-treated P23H rats (60 ± 2.5 cones/mm and 109 ± 2 cones/mm, respectively; ANOVA, Bonferroni’s test, *P* < 0.001, *n* = 6 in both cases; Fig. 3e).

**Fig. 4:**
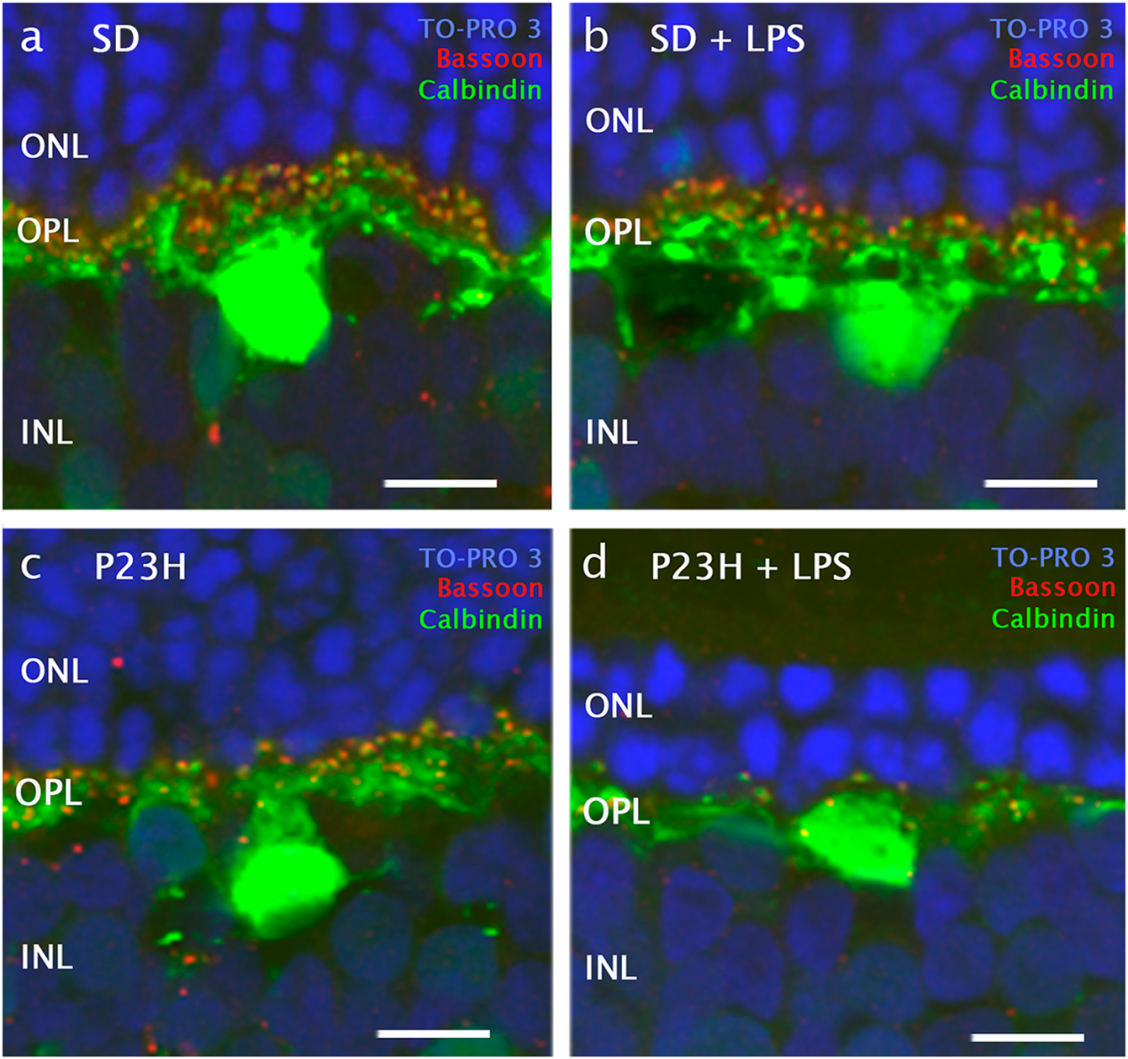
Effect of LPS on synaptic connectivity between photoreceptors and horizontal cells. High magnification confocal images of retinal sections showing synaptic contacts and dendritic arborization of representative horizontal cells after immunostaining with antibodies for horizontal cells (calbindin, green) and synaptic ribbons (Bassoon, red) in SD **a**, **b** and P23H rats **c**, **d**, administered with vehicle **a**, **c** or LPS **b**, **d**. Nuclei were stained with TO-PRO 3 (blue). INL: inner nuclear layer, OPL: outer plexiform layer, ONL: outer nuclear layer. Scale bar 10μm.

Given the significant decrease in the number of photoreceptor rows in P23H rats administered LPS, synaptic connections were assessed in the retina of dystrophic and normal animals. The analysis of retinal sections immunostained with polyclonal rabbit anti-calbindin antibodies showed no significant differences in the average number of horizontal cells between P23H or SD rats administered LPS or vehicle (Supplementary material 1). However, in LPS-injected P23H rats the dendritic arborization of horizontal cells was clearly degenerated as compared with vehicle-injected P23H rats (Fig. 4). Numerous Bassoon-immunopositive spots were observed at the INL level in P23H animals, regardless of whether they were administered LPS. By contrast, few synaptic ribbons were observed at the OPL in P23H rats injected with LPS (Fig. 4d), thus revealing that presynaptic contacts between photoreceptors and bipolar/horizontal cells were reduced in these animals.

**Fig. 5:**
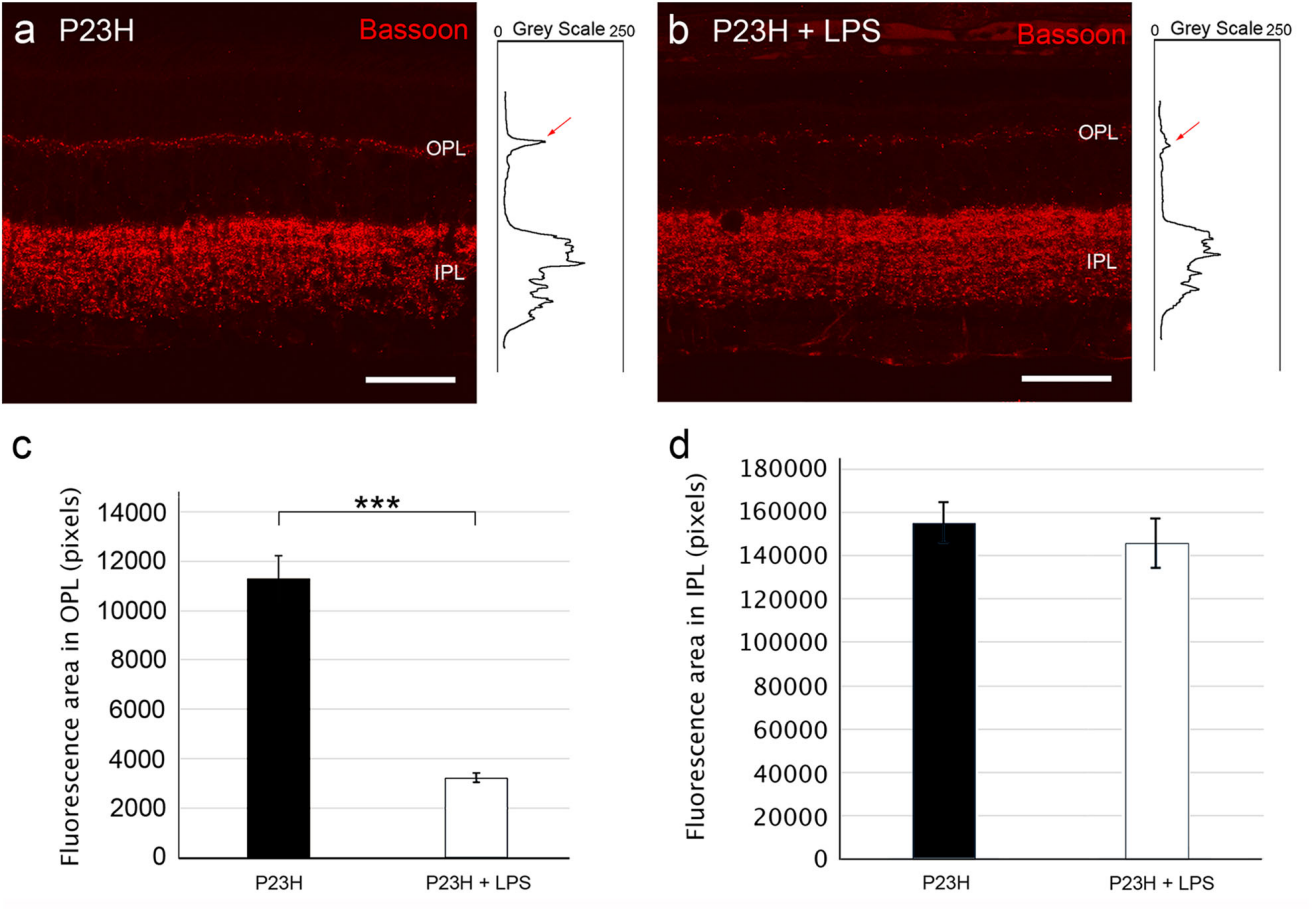
Effect of LPS on synaptic connectivity. **a**, **b** Representative vertical retinal sections showing synaptic ribbons immunostained with anti-Bassoon antibodies (red) from vehicle-injected **a** and LPS-injected **b** P23H rats. Right inserts show the profile plots of mean gray intensity for each horizontal line of images of the retina showing Bassoon immunofluorescence. **c**, **d** Quantification of the differential expression of Bassoon in the outer **c** and the inner plexiform layers **d** of P23H rats. Data are represented as mean values of fluorescence in areas close to the optic nerve ± SEM,* n* = 6. ***P < 0.001; ANOVA, Bonferroni’s test. IPL: inner plexiform layer, OPL: outer plexiform layer. Scale bar 40μm.

To quantify the differential expression of Bassoon, we obtained the profile plots of mean gray intensity in each horizontal line of images of the retina showing Bassoon immunofluorescence (Figs. 5a, b). As it is shown in Fig. 5c, the mean area of the profiles obtained at the OPL level in vehicle-injected P23H rats were significantly higher (3.5-fold greater) than those obtained in P23H rats administered LPS (11200 ± 500 pixels and 3500 ± 150 pixels, respectively; ANOVA, Bonferroni’s test, *P* < 0.001, *n* = 6 in both cases). No significant differences were observed at the IPL (Fig. 5d, 15500 ± 700 pixels and 14500 ± 850 pixels, respectively). In the SD rat groups, we did not find any significant differences in the fluorescent area of either plexiform layer (data not shown).

### The effects of systemic LPS administration on retinal microglial number and activation state

**Fig. 6:**
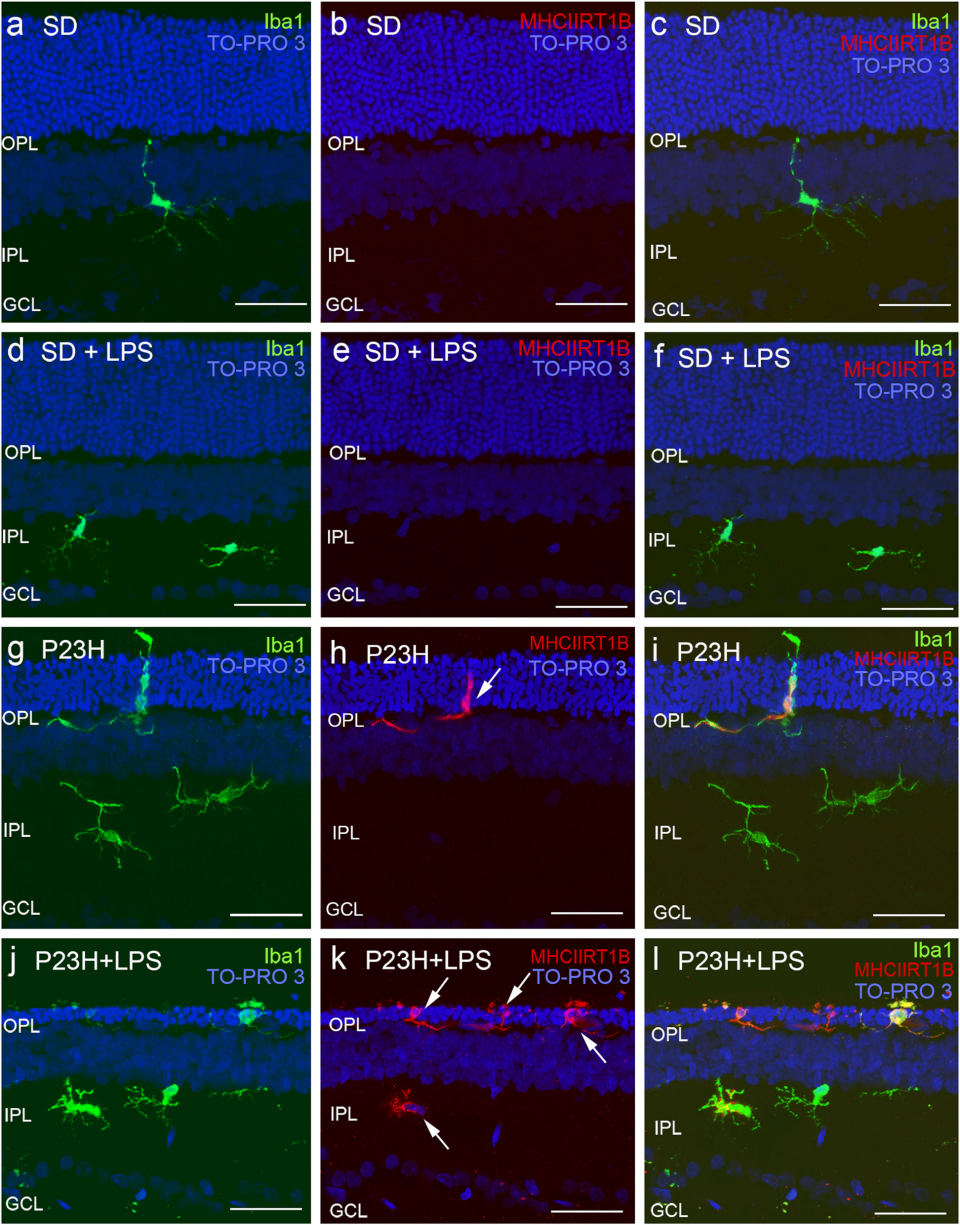
Effect of LPS on retinal microglia in SD and P23H rats. Representative vertical retinal sections immunostained with antibodies against Iba1 (green) and MHC-II (red) in vehicle- and LPS-injected SD rats **a**–**f**, or vehicle- and LPS-injected P23H rats **g**–**l**. Arrows point microglia MHC-II-immunopositive cells. Nuclei were stained with TO-PRO (blue). All images were taken in the central retina. GCL ganglion cell layer, IPL inner plexiform layer, OPL outer plexiform layer. Scale bar 40μm

**Fig. 7:**
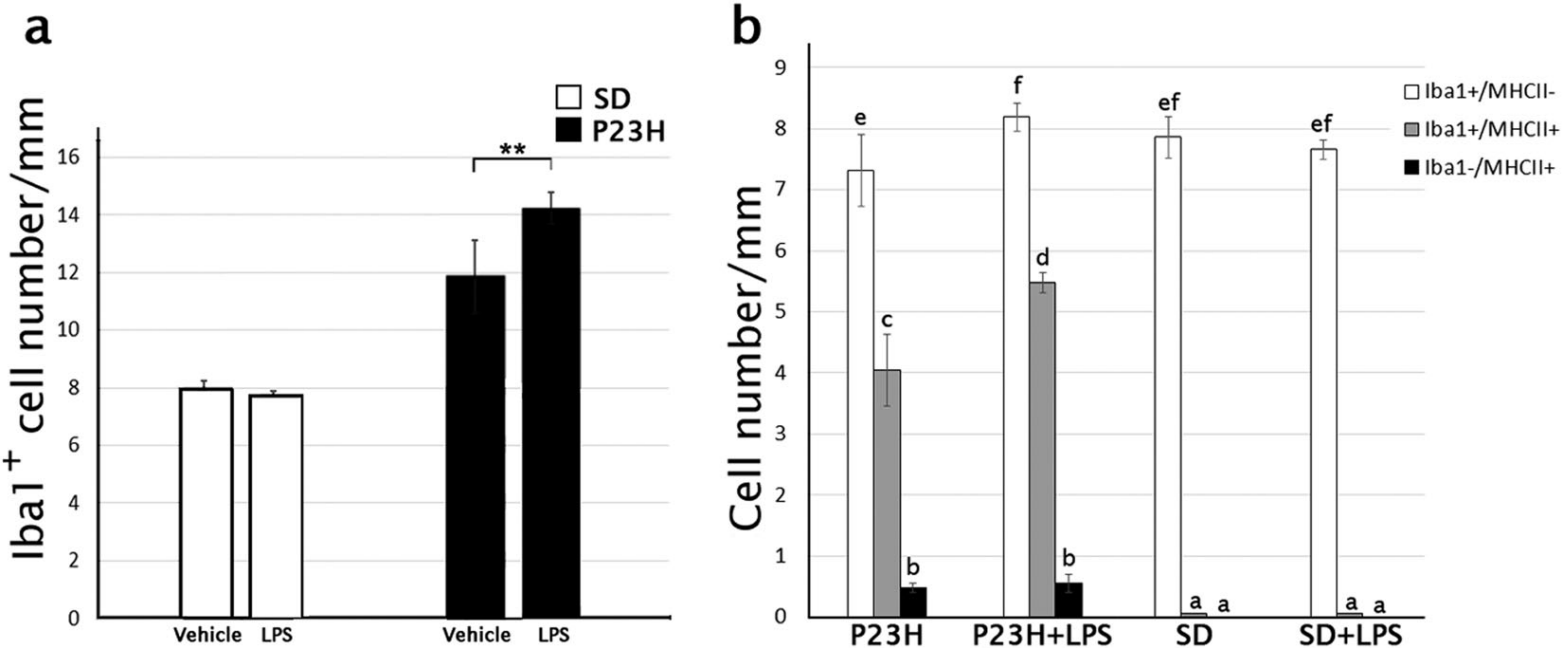
Effect of LPS on microglia cell number assessed by immunohistochemistry. Quantification of microglial cells in retinas from SD and P23H rats injected with vehicle or LPS after immunostaining with anti-Iba1 and anti-MHC-II antibodies. The figure shows the average of total Iba1-immunopositive cells **a** and the average of microglial cells showing the phenotypes Iba1+/MHC-II−, Iba1+/MHCII+ and Iba1−/MHC-II+ **b**. Data are presented as mean values ±SEM, n = 6. **P<0.01; ANOVA, Bonferroni’s test. Different letters indicate statistical significance

In SD rat retinas, Iba1+ cells were distributed in the inner and outer plexiform and ganglion cell layers (Fig. 6a) and showed negligible immunoreactivity against MHC Class II antibodies (Figs. 6b, c). In these animals, LPS injection did not induce any differences in the morphology, activation state, density, or distribution of retinal microglial cells (Figs. 6d–f). In P23H retinas, microglial cells were seen in all retinal layers, and were present in greater numbers in IPL and OPL (Figs. 6g–i), in a density higher than that observed in SD rats (11.8 ± 1 Iba1+ cells/mm and 7.7 ± 0.5 Iba1+ cells/mm, respectively; ANOVA, Bonferroni’s test, *P* < 0.01, *n *= 6, in both cases; Fig. 7a). Moreover, P23H rat retinas presented ameboid Iba1+ cells, some of them exhibiting the activation marker MHC-II (Figs. 6g–i). LPS injection in P23H rats increased the quantity of Iba1+ cells, many of them showing ameboid shape and expressing MHC-II (Figs. 6j-l). Quantitative analysis of the retinal sections showed that in LPS-injected P23H rats the relative quantity of Iba1+/MHC-II− cells was 12% higher than that observed in vehicle-injected P23H rats (ANOVA, Bonferroni’s test, *P* < 0.05; Fig. 7b). The number of double immunoreactive cells Iba1+/MHC-II+ was also significantly higher in LPS-injected P23H animals when compared with P23H rats (35% more; ANOVA, Bonferroni´s test, *P* < 0.001; Fig. 7b).

**Fig. 8:**
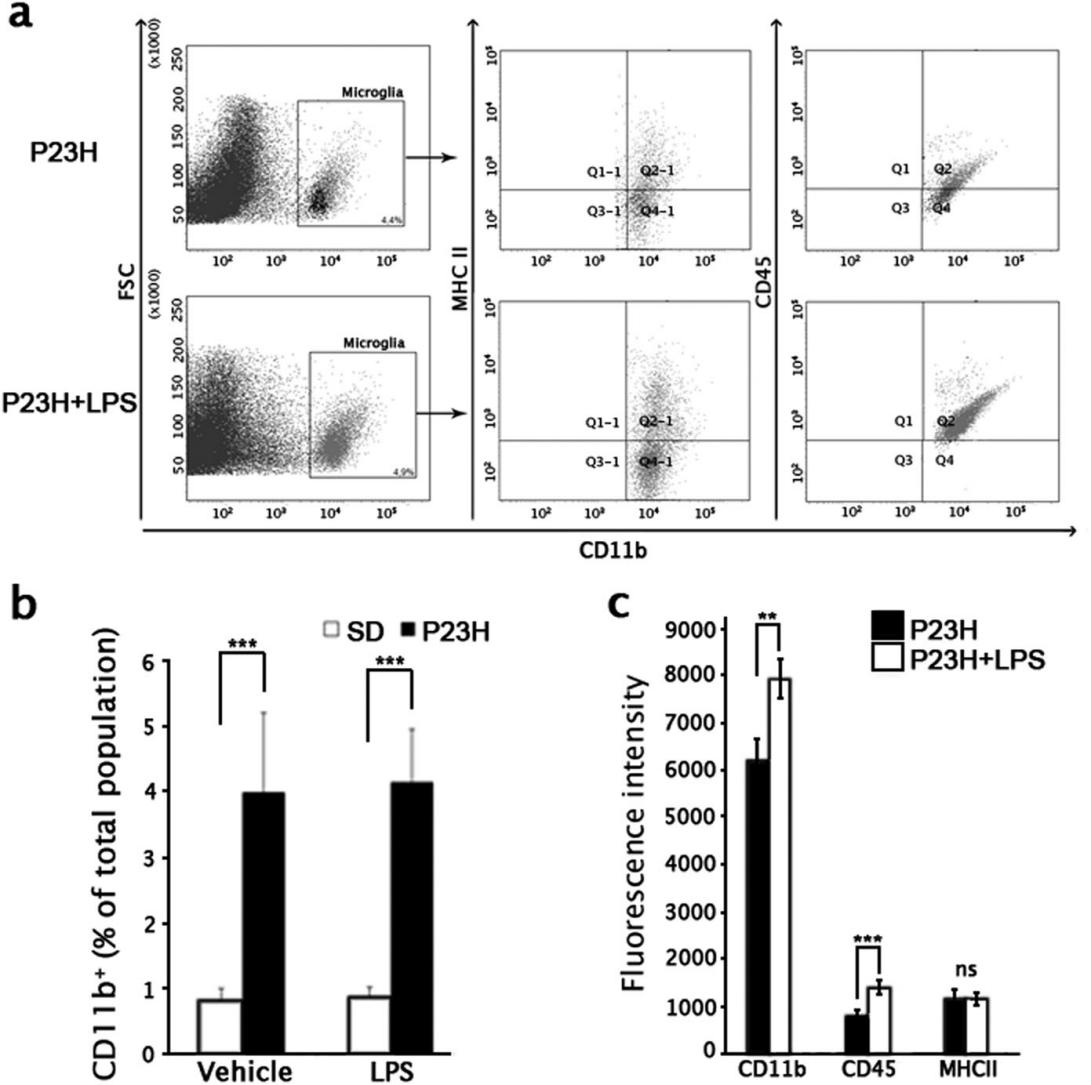
Effect of LPS on retinal microglial cell number and activation state, assessed by flow cytometric analysis. **a** Representative forward scatter (FSC) vs. CD11b dot plots, in which CD11b-positive cells are gated and double dot plots showing MHC-II vs. CD11b and CD45 vs. CD11b of CD11b-immunopositive cells. Each dot plot is representative of a minimum of four independent replicates. In all, 10^6^ events were recorded per sample and 500,000 events are represented. The percentage of CD11b-immunopositive population is indicated. **b** Mean values of CD11b-immunopositive population in vehicle-injected and LPS-injected SD and P23H rats. **c** Histograms representing mean fluorescence intensity values of the CD11b-positive population labeled with anti-CD45, anti-MHC-II, and anti-CD45 antibodies (10^6^ cells were analyzed in each assay) in both P23H experimental groups. Data are presented as mean values ± SEM. **P < 0.01, ***P < 0.001; Student’s *t*-test

In addition to the morphological and quantitative analysis of retinal sections, retinal cells expressing the microglia marker CD11b were identified by flow cytometry (Figs. 8a, b) and MHC-II and CD45 expression was measured to determine their level of activation (Figs. 8a, c). SD rats showed no significant differences in the number of retinal CD11b+ cells between LPS- and vehicle-injected animals (0.8 ± 0.2% in vehicle-injected vs. 0.9 ± 0.2% in LPS-injected animals, Fig. 8b). Between these groups, the amount and distribution of different phenotypes of microglial cells also did not show any differences. Otherwise, P23H retinas showed an increased CD11b+ microglial population (4.0 ± 1.2%) with respect to SD rat retinas (Student’s *t*-test, *P* < 0.001; Fig. 8b). The quantity of CD11b+ cells was not significantly different between P23H rats injected with vehicle or LPS, but the fluorescence intensity for CD11b and CD45 was significantly higher in LPS- injected vs. vehicle-injected P23H rats (Student’s *t-*test, *P* < 0.01 and *P* < 0.001, respectively; Fig. 8c).

### The effects of systemic LPS administration on astrocytes and Müller cells

**Fig. 9:**
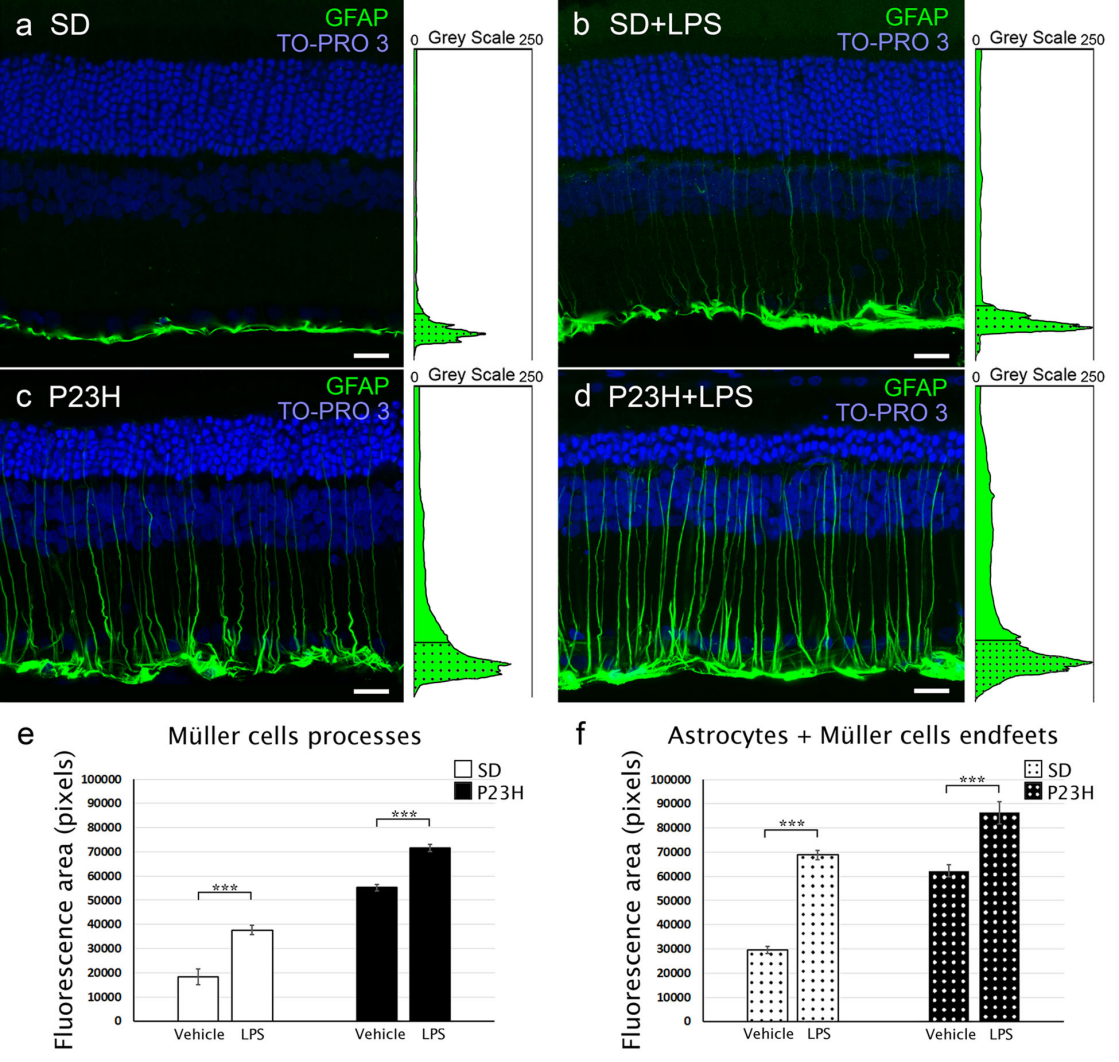
Effect of LPS on retinal Müller cells and astrocytes. **a**, **b** Representative vertical retinal sections from vehicle-injected **a**, **c** and LPS-injected **b**, **d** SD **a**, **b** and P23H **c**, **d** rats immunostained with anti-GFAP antibodies (green). Nuclei were stained with TO-PRO (blue). Note the reactive changes in astrocytes and Müller cells associated with LPS administration in both SD and P23H rats. **e**, **f** Quantification of GFAP immunostaining in the Müller cell processes **e** and the inner margin of the retina **f**, colocalizing with astrocytes and end feet of Müller cells. Data are represented as mean values of fluorescence in pixels ± SEM, *n* = 4 in each condition. ***P < 0.001; ANOVA, Bonferroni’s test. Scale bar 20μm

The expression of GFAP in healthy rat retinas is restricted to astrocytes within the inner retina^3^. However, under many pathological conditions or retinal damage, Müller cells are found to express GFAP^3,27^. Using anti-GFAP antibodies, we assessed the response of Müller cells and astrocytes to LPS treatment (Fig. 9). In vehicle-injected SD rats, GFAP immunoreactivity was virtually limited to the inner margin of the retina, colocalizing with astrocytes, whereas P23H rat retinas also showed GFAP immunoreactivity in Müller cells, which exhibited a marked labeling throughout the entire cell. In both strains, SD and P23H rats, LPS injection triggered an increase in GFAP immunoreactivity in the Müller cell processes as compared with vehicle-injected animals (37652 ± 2000 pixels and 18296 ± 3100 pixels, respectively, in SD animals and 71655 ± 1500 pixels and 55185 ± 1220 pixels, respectively, in P23H animals; ANOVA, Bonferroni’s test, *P* < 0.001, *n* = 4 in both cases; Figs. 9a–e). LPS treatment also triggered an increase in GFAP immunofluorescence in the inner margin of the retina, colocalizing with astrocytes and end feet of Müller cells (68834 ± 1900 pixels and 29613 ± 1500 pixels, respectively, in SD animals and 86308 ± 4500 pixels and 62251 ± 2600 pixels, respectively, in P23H animals; ANOVA, Bonferroni’s test, *P* < 0.001,* n* = 4 in both cases; Fig. 9f).

### The effects of systemic LPS administration on the retinal expression of inflammation- and apoptosis-related genes

**Fig. 10:**
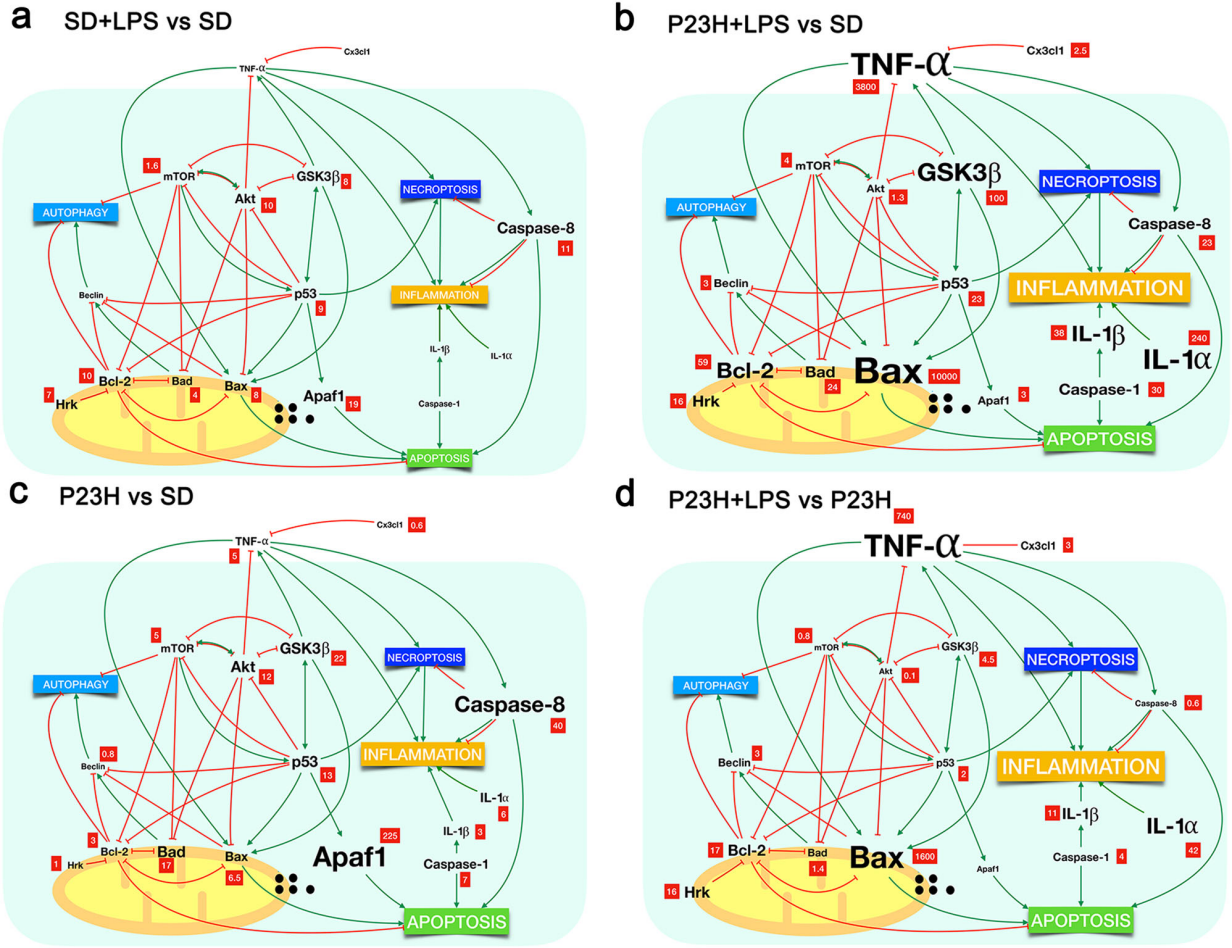
Schematic view showing relative expression levels of cell mediators in our experimental conditions and the interactions between and their effects on autophagy, necroptosis, inflammation, and apoptosis as described by others (see text for references). **a**–**c** Relative expression (fold change, in red squares) of mRNA in the retina of LPS-injected SD rats **a**, LPS-injected P23H rats **b** and vehicle-injected P23H rats **c**, compared with the expression levels found in vehicle-administered SD rats. **d** Normalized expression of mRNA in LPS-injected P23H rats, compared with that found in vehicle-administered P23H rats. The relative size of the letters represents the relative level of gene expression

We analyzed the mRNA expression levels of genes related to apoptosis and inflammatory processes by quantitative RT-PCR, as well as genes involved in regulatory pathways. Table 1 shows normalized relative mRNA levels (fold change) of the selected genes in the retina of each experimental group, compared with the expression level found in vehicle-injected SD rats. Systemic LPS injection in SD rats increased the retinal expression of *Caspase-8* (11-fold) and the apoptosis-related genes *Bad* (4), *Bax* (8), *Hrk* (7), *Bcl-2* (10), *Apaf-1* (19) and *p53* (9). In the retina of these animals, we also detected increased expression levels of *GSK3β* (8), altogether with *Akt* (10) and *mTOR* (1.6) (Fig. 10a). In vehicle-injected P23H rats, we saw increased retinal expression of the inflammation-related genes *TNF-α* (5), *IL-1α* (6), *IL-1β* (3), *Caspase-1* (7) and *Caspase-8* (40), and also of the regulatory genes *GSK3β* (22), *Akt* (12), and *mTOR* (5). In this experimental group, the retinal expression levels of apoptosis-related genes were: *Bad* (17), *Bax* (6.5), *Hrk* (1), *Bcl-2* (3), *Apaf-1*(225), and *p53* (13), whereas *CX3CL1* levels were reduced (0.6) (Fig. 10c). In dystrophic P23H rats, the systemic injection of LPS induced greater changes in retinal mRNA expression than that observed in SD rats, affecting genes related to both inflammation and apoptosis pathways: *Bad* (24), *Bax* (10000), *Hrk* (16), *Bcl-2* (59), *Apaf-1*(3) and *p53* (23), *CX3CL1* (2.5); *TNF-α* (3800), *IL-1*α (240), *IL-1β* (38), *Caspase-1* (30), and *Caspase-8* (23). The expression levels of regulatory genes were *GSK3β* (100), *Akt* (1213) and *mTOR* (4) (Fig. 10b). When comparing the expression levels of these genes in P23H LPS-injected rats with P23H vehicle-injected rats, the relative expression levels in the former were: *Bad* (1.4), *Bax* (1600), *Hrk* (16), *Bcl-2* (17), *p53* (2), *CX3CL1* (3); *TNF-α* (740), *IL-1β* (42), *IL-1β* (11), *Caspase-1* (4), *Caspase-8* (0.6); *GSK3β* (4.5), *Akt* (0.1), and *mTOR* (0.8) (Fig. 10d).

**Table 1:** Effect of LPS on the expression of cell mediators

Relative expression levels-qRT-PCR										
Inflammation-related genes	SD	SD + LPS	95% CI	*P*-value	P23H	95% CI	*P*-value	P23H + LPS	95% CI	*P*-value
TNF-α	1				5	(0.73, 7.50)	ns	3800	(983.72, 4377.68)	*
IL-1α	1				6	(0.00001, 8.78)	*	240	(23.12, 324.18)	**
IL-1β	1				3	(0.55, 4.93)	*	38	(8.36, 43.37)	***
Casp-1	1				7	(1.97, 11.72)	***	30	(4.51, 66.28)	***
Casp-8	1	11	(0.00001, 24.23)	***	40	(0.00001, 82.74)	ns	23	(0.00001, 58.43)	ns
CX3CL1	1				0.6	(0.13, 1.63)	***	2.5	(0.09, 3.80)	***
Beclin	1				0.8	(0.28, 1.06)	***	3	(0.61, 4.53)	***
Apoptosis-related genes	SD	SD + LPS	95% CI	*P*-value	P23H	95% CI	*P*-value	P23H + LPS	95% CI	*P*-value
Bad	1	4	(2.72, 5.57)	***	17	(7.20, 26.96)	*	24	(0.00001, 71.18)	*
Bax	1	8	(0.00001, 19.45)	***	6.5	(0.00001, 18.29)	**	10,000	(0.00001, 102544.04)	**
Hrk	1	7	(0.00001, 15.48)	***	1	(0.00001, 2.39)	ns	16	(0.00001, 92.52)	ns
Bcl-2	1	10	(0.00001, 20.16)	***	3	(0.00001, 7.68)	ns	59	(0.00001, 169.61)	ns
Apaf-1	1	19	(0.00001, 57.07)	ns	225	(0.00001, 787.25)	*	3	(0.00001, 23.01)	*
p53	1	9	(1.94, 16.44)	***	13	(0.56, 24.83)	ns	23	(0.00001, 84.41)	ns
Regulation-related genes	SD	SD + LPS	95% CI	*P*-value	P23H	95% CI	*P*-value	P23H + LPS	95% CI	*P-*value
GSK3β	1	8	(0.00001, 15.69)	**	22	(0.00001, 52.09)	***	100	(0.00001, 250.22)	**
Akt	1	10	(0.00001, 22.47)	**	12	(0.00001, 33.75)	*	1.3	(0.00001, 9.34)	ns
mTOR	1	1.6	(0.32, 2.99)	*	5	(0.45, 9.81)	ns	4	(0.00001, 8.36)	ns

mRNA quantification of genes related to apoptosis and inflammation pathways and the Akt, GSK3β and mTOR signaling pathways mRNA expression was analyzed by quantitative RT-PCR. Values represent the normalized relative expression (in fold) of mRNA in the retina of each experimental group, as compared with the expression levels found in vehicle-administered SD rats

*P < 0.05, **P < 0.01, ***P < 0.001; Student’s *t*-test of the replicate 2^(-ΔΔCT) values for each gene in the vehicle and LPS groups

## Discussion

The potential influence of systemic pathologies in the progression of retinal neurodegenerative diseases is a crucial issue to be explored due to its expected influence on the prognosis and therapy. Our main objective was to assess whether peripheral inflammatory conditions can worsen the progression of neurodegeneration in an animal model of RP. In order to mimic the effect of a mild chronic condition, we chronically administered low doses of LPS, which did not induce physiological or morphological changes in healthy retinas.

### LPS-induced systemic inflammation aggravates morphological and physiological disturbances in dystrophic retinas

Compared with healthy rat retinas, P23H transgenic rat retinas showed a decreased number of photoreceptors and synaptic contacts, as well as lower ERG responses. Hereditary retinal degeneration was accompanied by microglial activation, evidenced by increased microglial density and higher expression levels of microglial activation markers. This is in agreement with previous findings showing that neurodegenerative retinal diseases are linked to chronic microglial activation and neuroinflammation^3,4,28,29^. Activated microglia mediate neuroinflammation by releasing several pro-inflammatory molecules including cytokines, chemokines, trophic factors, and small molecules, which promote and perpetuate the inflammatory response, potentially leading to neurodegeneration^3,10,30–32^.

As a result of repeatedly administering low doses of LPS to P23H degenerating retinas, a further dramatic decrease in the quantity of photoreceptor cells and synaptic connectivity in the OPL was observed, in agreement with the lower ERG responses observed in this experimental group. Specifically, it has been demonstrated that systemic inflammation induced by LPS increases neuronal apoptosis in the brain of an animal model of prion disease^33^. Hippocampal-dependent learning deficits are triggered by LPS-induced peripheral inflammation in elderly rats^34^, and repeated challenges with LPS cause increased Microtubule associated protein (MAP, Tau) hyperphosphorylation in a transgenic model of Alzheimer’s disease^35^. SD control rats administered with the same doses of LPS failed to show retinal changes at either a structural or physiological level.

### LPS-induced systemic inflammation triggers microglial activation and gliosis in dystrophic retinas

The LPS-induced microglial activation observed in P23H rat retinas is in agreement with our previous works and those by others, showing that systemic infections caused by cytomegalovirus^15^ or *Candida albicans*^16^, as well as peripheral insults^17^ are capable of triggering microglial activation and increasing the quantity of microglial cells in the retina. The higher number of Iba1-positive cells in the retina of LPS-injected P23H rats could be caused by migration of inflammatory macrophages, myeloid precursors, or inflammatory monocytes, or by the proliferation of retinal microglia, as these cell types are able to proliferate in situ once they have differentiated^36^. In a previous work, we evidenced the presence of proliferative Iba1+ cells in P23H rat retinas, but not in healthy control rats, suggesting that increases of Iba1-positive cells in P23H rats is accomplished, at least partially, by the in situ proliferation of resident microglia^4^.

In this study, we show that LPS injection induces an increase in GFAP immunoreactivity in the Müller cell processes in the inner margin of the retina of P23H rats. A hallmark of gliosis is the upregulation of intermediate filament proteins in glial cells, including GFAP. Thus, our results suggest that low-dose LPS treatment exacerbates reactive gliosis in astrocytes and Müller cells.

### Systemic inflammation exacerbates inflammatory and apoptotic pathways in dystrophic retinas

At the molecular level, low-dose LPS treatment in SD rats induced upregulation of several important apoptosis-related genes, involved in both death and survival signals (*Bcl-2*, *Bax*, *Hrk*, *p53*, *Apaf-1*, and* Caspase-8*), which can be associated with an early cellular response. In this experimental group, we could not detect any increased expression of inflammation-related genes. This result is consistent with the normal appearance of these retinas, and probably due to the low doses of LPS we used. As we did not detect any functional nor morphological change in the retinas, we assume that retinal cells were able to maintain cellular homeostasis. P23H rat retinas showed increased expression levels of several inflammation-related genes (*Tnf-α, Il-1α*, *Il-1β, Caspase-1,* and* Caspase-8*) in agreement with the higher inflammatory state of the degenerating retina that is observed during all the degenerative process, compared with healthy retinas^4^. This result also agrees with the high levels of pro-inflammatory cytokines present in certain neurodegenerative diseases, such as Parkinson’s^6,37^, Alzheimer’s^7,8^, and Huntington’s diseases or amyotrophic lateral sclerosis^9^. In P23H rat retinas, we also detected increased levels of expression of genes associated with apoptosis (*p53, Bcl-2, Bad, Bax*, and *Apaf-1*), in concordance with the enhanced rate of apoptosis observed in this work and in previous published studies^38^. In P23H rats, LPS treatment induced a drastic further increase in the expression of inflammation- and apoptosis-related genes, which agrees with other authors’ results showing that systemic administration of LPS, even in a single dose, causes increased microglial activation, inflammatory exacerbation, and enhanced neurodegeneration through increased production of TNF-α, IL-1β, and IL-6 in the brain^39,40^. In P23H model of retinal degeneration, an increased expression of Tnf-α, Il-1α, and Il-1β could, at least in part, explain the increased loss of photoreceptors observed in LPS-injected P23H rats^3,10,30–32,41–43^. In LPS-injected P23H rats, we found further upregulation of the *Caspase-1* gene, pointing to a scenario of even greater apoptosis and inflammation^44–46^.

In agreement with the accepted main role of p53 in neuroinflammatory processes in neurodegeneration^47^, in our experimental conditions, the transcription factor p53 also appears as a key factor in the neurodegenerative process. *p53* expression was enhanced in all the conditions tested, with higher increases in P23H rat retinas than in LPS-injected SD rat retinas, and even higher expression levels in the retinas of LPS-injected P23H rats. Hence, in our experiments, the response obtained with repeated low doses of LPS reinforce the idea that a chronic inflammatory peripheral state does affect the degenerative process by increasing the expression of inflammation- and apoptosis-related markers.

Concordant to its activity as an early modulator in damaged tissues^48–51^, Caspase-8 expression levels were increased in all our experimental groups (SD LPS-injected, P23H vehicle-injected, and P23H LPS-injected).

The activation of TLR4 by LPS can participate in cell apoptosis through the phosphatidylinositol 3 kinase/AKT/GSK3β signaling pathway^52^. In our experiments, LPS administration in low doses to SD rats provoked an imbalance between pro-death and pro-survival signals can be determinant for the final outcome of the cell. Additionally, the Akt, Glycogen Synthase Kinase 3 beta (GSK3β), and Mammalian Target of Rapamycin (mTOR) signaling pathways have been implicated in the final outcome of threatened cells^52–59^. Inhibitors of Glycogen Synthase Kinase 3 beta (GSK3β) provide potential therapeutic strategies to control inflammation^60^, and are thus drugs that may potentially be used for the treatment of RP^61^.

**Fig. 11:**
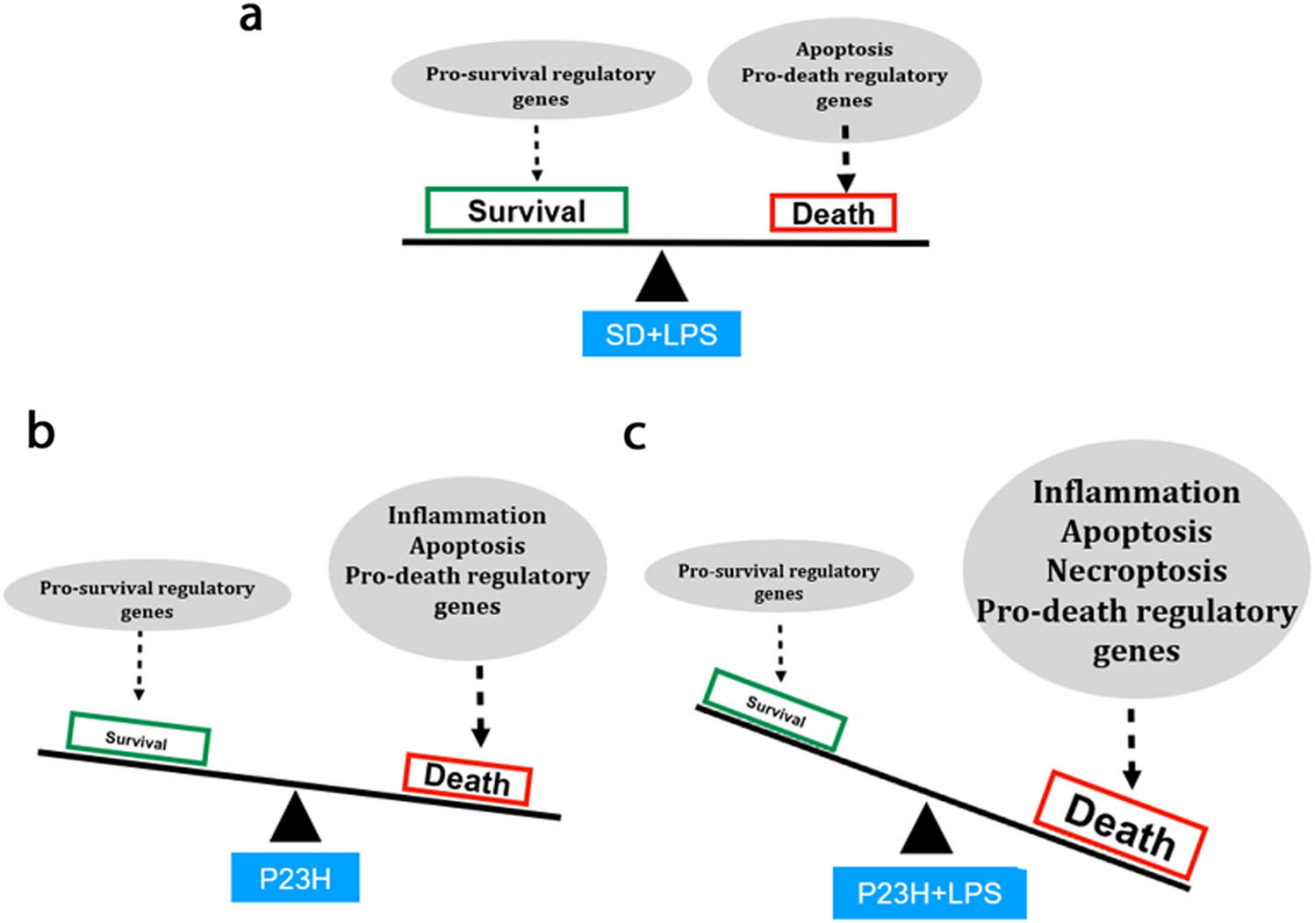
Schematic representation of the hypothetical contribution of death pathways to the outcome of the cell in different noxious situations in animal retinas: **a** chronic administration of low doses of LPS (from P20 to P60) to SD healthy rats, **b** genetically determined retinal dystrophy (P23H rat model of RP), and **c** chronic administration of low doses of LPS to the genetically determined dystrophic rats (P23H model). Our results indicate that in healthy retinas, low doses of LPS induce expression changes in pro- and anti-death signals, as well as in apoptosis-related transcripts, although no signs of cell death are observed. In P23H rat retinas, increased expression levels of genes related to apoptosis and inflammation and pro-death signals alter the balance between pro-survival and pro-death signals and influence the death scenario observed at P60. In this model of degeneration, the administration of low doses of LPS probably induces an increase in necroptotic cell death, together with insufficient autophagic mechanisms that would worsen the retinal degenerative process and contribute to the exacerbation of the morphological and functional damage, concordant with the morphological worsening and loss of function observed

Our results revealed that LPS administration induced increased expression levels of *mTOR* in the retina of SD rats, in agreement with other works that attribute to *mTOR* a main role in the inflammatory effects of LPS^58,62,63^. The expression levels of *mTOR* were higher in both P23H rat groups, which could contribute to the increased degeneration observed in these animals, as previously described for retinal degenerative processes^59^. We hypothesize that the effects of LPS may alter the balance between pro-survival and pro-death signals and exacerbate the cell death processes (Fig. 11).

### Systemic LPS can modify death pathways contribution to the degenerative process in dystrophic retinas

In this death scenario, the contribution of other death pathways as necroptosis and autophagy should be taken into account. When compared with vehicle-injected P23H rat retinas, the retinas of P23H rats injected with LPS showed an enhanced death stimulus, with dramatically increased expression levels of *Tnf-α* and *p53*^51,64^. As both apoptosis and necroptosis can be triggered simultaneously in the retina^65,66^, these data, together with the downregulation of *Caspase-8*, and the reduced expression of the pro-apoptotic *Apaf-1* in these animals, leads us to hypothesize that LPS trigger the activation of necroptosis pathways in the retina of dystrophic rats. Necroptosis cell death would contribute to the augmented inflammatory response in the retina of these animals and to the worsening of the morphological and functional activity. This hypothesis agrees with prior studies showing that retinal microglia undergo RIP1/3-mediated necroptosis in rd1 mouse model of retinal neurodegeneration and also after an acute retinal neural injury^67^. Necroptotic microglia release large amounts of pro-inflammatory cytokines, such as TNF-α, in response to LPS or oxidative stress, triggering neuroinflammation and, consequently, exacerbating retinal damage and disease progression^67^.

Previous studies showed that LPS treatment can promote autophagy in microglia and macrophages^68,69^. In our experiments, LPS administration to P23H dystrophic rats induced increased levels of *Beclin-1* and *Bad* expression and reduced expression levels of *mTOR*. These results suggest a higher degree of autophagy processes^70^. But the higher levels of *Bax, Bcl-2*, and *p53*^31,70–76^ seem to point to the fact that, despite the increased pro-autophagy markers apoptotic and inflammatory state in these animals is dampening the activation of appropriate autophagy mechanisms. Autophagy dysfunction has been related to a number of neurodegenerative diseases, such as Alzheimer’s or Parkinson’s diseases, among other neuron-affecting pathologies^7,9,69,77,78^.

In the degenerating retinas of P23H rats treated with LPS, the insufficient autophagy together with the promotion of necroptotic cell death would worsen the degenerative process and contribute to the exacerbation of the morphological and functional damage.

## Conclusions

All these data indicate that administering low doses of LPS to dystrophic P23H rats exacerbates inflammatory and apoptotic states, accelerates the loss of cone photoreceptors and retinal functionality, and triggers the worsening of negative symptoms of the disease. Given that LPS administration emulates the noxious influence of a peripheral inflammatory condition, these results are particularly important for patients that suffer from ocular neurodegenerative diseases such as glaucoma, diabetic retinopathy, RP, or macular degeneration. According to these results, peripheral damage, such as a systemic infection or chronic inflammatory process, could accelerate the progression of the disease and should be considered when selecting an appropriate therapy to revert, block or slow-down the degenerative process.

Neurodegenerative diseases share common pathogenic mechanisms, including neuroinflammation. In this sense, the activation of microglial cells has been extensively reported in both retinal and brain diseases^3,79^. On the other hand, it has been previously shown that systemic conditions can worsen and exacerbate the symptoms of chronic neurodegeneration in degenerative diseases as Parkinson’s and Alzheimer’s disease, accelerating the progression of the disease^40,80–83^. Therefore, our results suggest a possible harmful effect of systemic inflammation on the progression of retinal degenerative diseases that could be extrapolated to brain neurodegenerative disorders, such as Parkinson’s or Alzheimer’s diseases.

## Electronic supplementary material

Supplemental material 1

Figure Legend
