# Supplementary figures and images for: Systemic inflammation induced by lipopolysaccharide aggravates inherited retinal dystrophy
Source: Cell Death Dis. 2018 Mar 2;9(3):350. doi: 10.1038/s41419-018-0355-x (PMC5834451; doi:10.1038/s41419-018-0355-x)

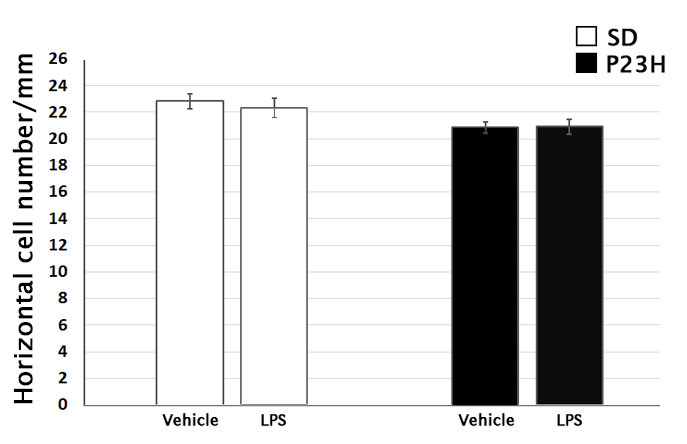

Supplement: Supplementary file 1 — Supplemental material 1 [file 41419_2018_355_MOESM1_ESM.tif]
