# Supplementary material for: Systemic inflammation induced by lipopolysaccharide aggravates inherited retinal dystrophy
Source: Cell Death Dis. 2018 Mar 2;9(3):350. doi: 10.1038/s41419-018-0355-x (PMC5834451; doi:10.1038/s41419-018-0355-x)
Supplement: Supplementary file 2 — Figure Legend [file 41419_2018_355_MOESM2_ESM.docx]

**Figure Legend**

Figure S1. Effect of LPS on horizontal cell number assessed by immunohistochemistry. Quantification of horizontal cells in retinas from SD and P23H rats injected with vehicle or LPS. The figure shows the average of total calbindin immunopositive cells. Data are presented as mean values ± SEM, n=6; ANOVA, *Bonferroni’s test*.
